# Prognostic Value of FGFR Gene Amplification in Patients with Different Types of Cancer: A Systematic Review and Meta-Analysis

**DOI:** 10.1371/journal.pone.0105524

**Published:** 2014-08-29

**Authors:** Jinjia Chang, Xinyang Liu, Shanshan Wang, Zhe Zhang, Zheng Wu, Xiaowei Zhang, Jin Li

**Affiliations:** Department of Medical Oncology, Fudan University Shanghai Cancer Center; Department of Oncology, Shanghai Medical College, Fudan University, Shanghai, China; Institute of Biomedicine, Finland

## Abstract

**Background:**

Fibroblast growth factor receptor (FGFR) gene amplification has been reported in different types of cancer. We performed an up-to-date meta-analysis to further characterize the prognostic value of FGFR gene amplification in patients with cancer.

**Methods:**

A search of several databases, including MEDLINE (PubMed), EMBASE, Web of Science, and China National Knowledge Infrastructure, was conducted to identify studies examining the association between FGFR gene amplification and cancer. A total of 24 studies met the inclusion criteria, and overall incidence rates, hazard risk (HR), overall survival, disease-free survival, and 95% confidence intervals (CIs) were calculated employing fixed- or random-effects models depending on the heterogeneity of the included studies.

**Results:**

In the meta-analysis of 24 studies, the prevalence of FGFR gene amplification was *FGFR1*: 0.11 (95% CI: 0.08–0.13) and *FGFR2*: 0.04 (95% CI: 0.02–0.06). Overall survival was significantly worse among patients with FGFR gene amplification: *FGFR1* [HR 1.57 (95% CI: 1.23–1.99); *p* = 0.0002] and *FGFR2* [HR 2.27 (95% CI: 1.73–3.00); *p*<0.00001].

**Conclusions:**

Current evidence supports the conclusion that the outcomes of patients with FGFR gene amplified cancers is worse than for those with non-FGFR gene amplified cancers.

## Introduction

The fibroblast growth factor receptor (FGFR) family comprises four main members (*FGFR1-FGFR4*) and encodes membrane tyrosine kinase receptors involved in signaling by interacting with fibroblast growth factors [1]. FGFR gene amplification is frequent in breast cancer, gastric cancer and lung cancer *etc*. In contrast to the activation of *FGFR3* and *FGFR4* by mutation [2], [3], amplification of *FGFR3* and *FGFR4* has been described only rarely in cancer and no data related to prognosis could be obtained. As a result, we mainly discuss *FGFR1* and *FGFR2* amplification in our present study.

*FGFR1* is one of the most commonly amplified genes in human cancer. Recently, *FGFR1* amplification has been demonstrated to be an independent negative prognostic factor in surgically resected squamous cell carcinoma of the lung [4]. Some of other types of cancer such as oral squamous carcinoma [5], esophageal squamous cell carcinomas [6], breast cancer [7]–[9] and pancreatic cancer [10] have also been reported to be associated with *FGFR1* amplification. *FGFR2*, the second most commonly amplified gene of the FGFR family, has been shown to be amplified in gastric cancer [11], [12], breast cancer [13], and non-small-cell lung cancer [14]. As a new candidate for a ‘driver gene’ in gastric cancer, *FGFR2*-targeted therapy has shown great potential in the treatment of gastric cancer [15], [16]. The aim of this study was to perform a systematic review and meta-analysis on the incidence of FGFR gene amplification, as well as the influence of *FGFR1* and *FGFR2* amplification on the outcomes of different types of cancers, and to provide an overview of the current status of FGFR gene amplification and cancer progression.

## Methods

### Literature search strategy

This analysis was performed in accordance with Preferred Reporting Items for Systematic Reviews and Meta-Analyses: the PRISMA Statement [17]. We searched the online databases MEDLINE (PubMed), EMBASE, Web of Science, and China National Knowledge Infrastructure up to August 2, 2013, without language limitations. For PubMed, the following contextual query language was used: (“FGFR” OR “fibroblast growth factor receptor”) AND (“cancer” OR “neoplasm” OR “carcinoma”). Reference lists of identified studies and reviews were also hand-searched.

### Study selection

Study eligibility was determined by two reviewers independently. Disagreements were solved by consensus. We included full papers and abstracts, without language restrictions, that: (i) studied FGFR gene amplification in any type of human cancers; (ii) measured FGFR gene amplification in human samples; and (iii) reported data necessary to calculate the incidence of FGFR gene amplification and/or HR on survival outcomes. Studies were excluded if they were: (i) reviews, case-only studies, or familial studies; (ii) lacking sufficient data for calculation of incidence and/or HR with 95% CIs; and (iii) duplication of previous publications or replicated samples.

### Data extraction and quality assessment

Data extraction was carried out by two reviewers independently, using a predefined form. Disagreements were resolved by discussion with a third reviewer. From each study, the following information was extracted: country of origin of the study, first author's name, year of publication, study population, FGFR gene amplification assessment methods, cut-off definition, and incidence of FGFR gene amplification with 95% CIs, HR for OS, and/or DFS with corresponding 95% CIs. In the studies that included cohorts of different ethnic populations, the data were collected separately and the data sets were recognized as independent studies. If the HRs and CIs were not reported, the total observed death events and the numbers of patients in each group were extracted to calculate HR and its variance indirectly [18]. In studies for which only Kaplan-Meier plots were available, data was extracted from the graphical survival plots. When both univariate analysis and multivariate analysis were reported to get the HR, the results of multivariate analysis, including other variables, were preferentially taken as they would be more accurate.

Study quality was assessed independently by the two reviewers using the following factors: (i) clear definition of the study population and the type of carcinoma; (ii) clear definition of the measurement method and the cut-off value of FGFR gene amplification; (iii) sample size larger than 10; and (iv) clear definition of the outcome assessment (if applicable). Any studies lacking any of these points were excluded from the final analysis.

### Statistical analysis

For the incidence of FGFR gene amplification, the incidences and 95% CIs were combined. For the survival analyses, HRs with 95% CIs were used to combine the pooled data. Heterogeneity was assessed by a Q-test. A fixed-effect model was used when there was no heterogeneity (*p*≥0.10) [19], otherwise a random-effect model was used [20]. For exploration of heterogeneity, subgroup analyses were performed based on cancer type, ethnicity, and assessment method. Sensitivity analyses were performed to assess the stability of the results, namely, a single study was deleted each time to reflect the influence of the individual data set on the results. Begg's funnel plots and Egger's tests [21] were used to assess publication bias. All the *p* values were two-sided, with *p*<0.05 considered statistically significant except for the Q-test. Statistical analyses were conducted using STATA version 11.0 (StataCorp LP, College Station, TX, USA) and Review Manager Version 5.1 (Copenhagen: The Nordic Cochrane Centre, The Cochrane Collaboration, 2011).

## Results

### Trail flow

**Figure 1:**
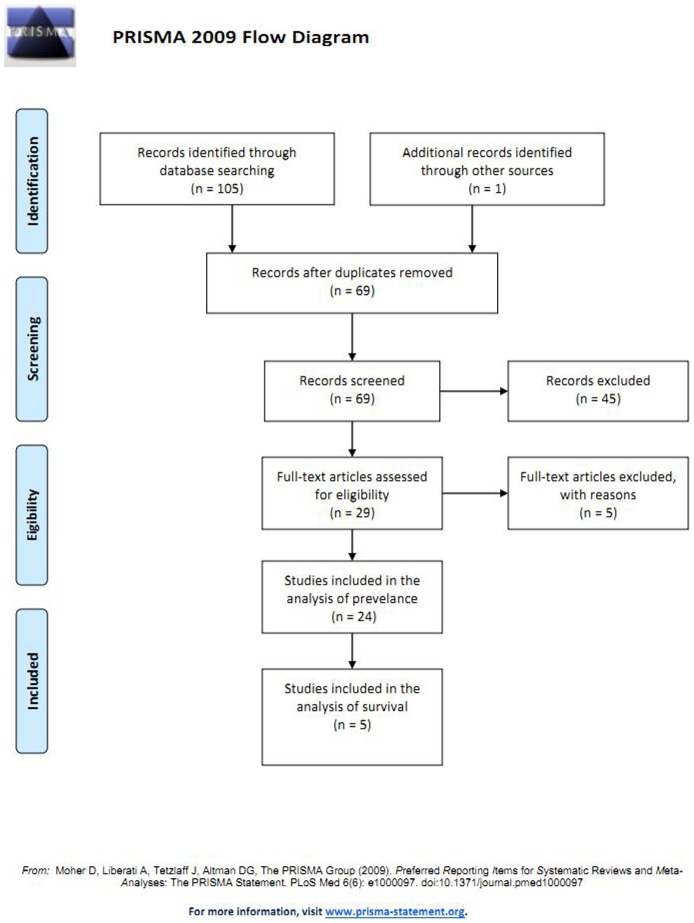
Flow diagram of the study selection process.

Figure 1 showed the results of the literature search. A total of 106 potentially relevant abstracts were found, and 24 studies were included in the analysis after screening. Most of the excluded abstracts were reviews or research with insufficient data.

### Characteristics of the studies

In this analysis, 4394 cases from 17 studies [4]–[10], [14], [22]–[30] were used to study *FGFR1* amplification and 2247 cases from 7 studies [11]–[14], [31]–[33] were used to investigate *FGFR2* amplification. For *FGFR1* amplification, 9 of 17 studies were in lung cancer, 4 studies were in breast cancer, and the other 4 studies were about oral and tongue squamous cell carcinoma, and oral squamous cell carcinoma. For *FGFR2* amplification, 5 of 7 studies were in gastric cancer, and the other 2 studies were in breast cancer and lung cancer. The main characteristics of the included studies were shown in Table S1. Additionally, prognostic data were obtained from 6 of 17 studies on *FGFR1* amplification and 3 of 7 studies (4 datasets) on *FGFR2* amplification.

### Method of evaluation *FGFR* amplification

Single-nucleotide polymorphism (SNP) array, quantitative polymerase chain reaction (qPCR), assay comparative genomic hybridization (aCGH), fluorescence in situ hybridization (FISH), chromogenic in situ hybridization (CISH) and silver in situ hybridization (SISH) [29] were used to determine FGFR gene amplification. FISH was the most commonly used method (18 of 24 studies). Most notably, the criteria for FGFR gene amplification were highly heterogeneous among different studies using FISH. For example, in some studies, *FGFR1/CEN8* greater than 2 [10], [29], [30], 2.2 [24], [25] and 4 [26], and *FGFR2/CEP10* greater than 2 [12], [32] were considered to be FGFR gene amplification (see Table S1). However, for the rest of the studies, the definition of FGFR gene amplification varied.

### Prevalence of FGFR gene amplification

**Figure 2:**
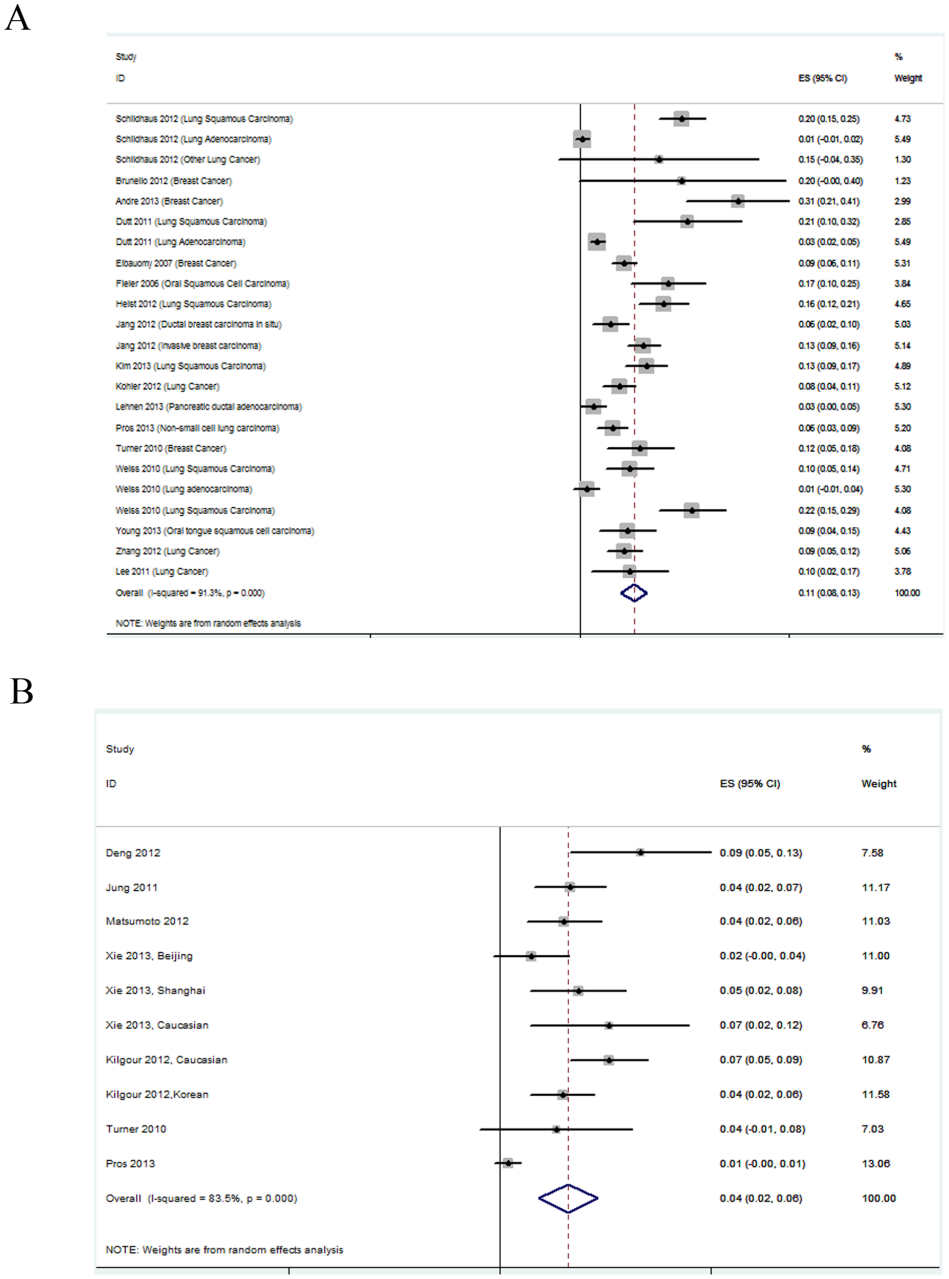
Forest plots describing the prevalence of *FGFR* amplification. (A) Analysis of the prevalence of *FGFR1* amplification. (B) Analysis of the prevalence of *FGFR2* amplification. The horizontal lines represent 95% CIs for estimating prevalence of FGFR gene amplification.(▪) Overall estimates of the effects.CI, confidence interval; ES, estimation.

The prevalence of *FGFR1* amplification in these studies ranged from 0 to 30.9%, partly reflecting the heterogeneity in the criteria for gene amplification. In the meta-analysis of 17 studies, the prevalence of *FGFR1* amplification was 0.11 [95% confidence interval (CI): 0.08–0.13] and large heterogeneity existed (*I^2^* = 91.3%; *p* = 0.000; Figure 2A). Subgroup analysis was stratified by cancer type, ethnicity, and methods, but the heterogeneity could not be reduced (Table S2). For *FGFR2* amplification, the prevalence in different studies was all under 10%. Six studies were assessed (Figure 2B) and the combined prevalence was 0.04 (95% CI: 0.02–0.06). The results also showed high heterogeneity (*I^2^* = 83.5%; *p* = 0.000). In addition, we checked the public Cancer Genome Atlas (http://cancergenome.nih.gov) for the prevalence of FGFR gene amplification. The results showed that *FGFR1* amplification occurred in 3.4% of 10,648 patients and *FGFR2* amplification occurred in 0.9% of 8352 patients. Consistent with our results, the amplifications were most commonly found in lung cancer (16.9%), breast cancer (13.4%), and gastric cancer (5.1%).

### Meta-analysis of FGFR gene amplification and cancer prognosis

**Figure 3:**
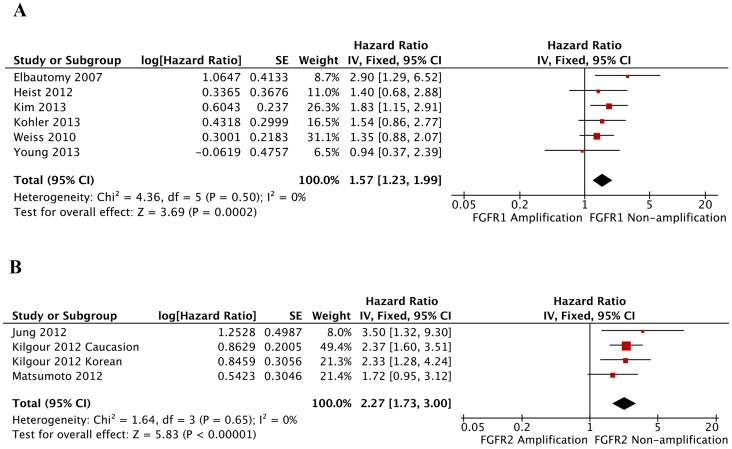
Forest plots of studies evaluating HR of overall survival, comparing high *FGFR* amplification and non-amplification. (A) Analysis of *FGFR1* amplification and overall survival in various cancers. (B) Analysis of *FGFR2* amplification and overall survival in gastric cancer. The horizontal lines represent 95% CIs for estimating HR of FGFR gene amplification versus non-amplification. (▪) Overall estimates of the effects. CI, confidence interval; HR, hazard ratio; IV, XXX; SE, standard error.

Pooled overall survival (OS) was used to illustrate FGFR gene amplification overall effect estimates for the studies containing prognostic data. Meta-analysis of FGFR gene amplification status and OS in a variety of cancers was performed; 1345 patients in 6 studies for *FGFR1* amplification and 1344 patients in 3 studies for *FGFR2* amplification were included. Notably, the patients in the analysis of *FGFR2* amplification were all gastric cancer patients. The results showed that the pooled hazard risks (HRs) were significant for both *FGFR1* [HR 1.57 (95% CI: 1.23–1.99); *p* = 0.0002] and *FGFR2* [HR 2.27 (95% CI: 1.73–3.00); *p*<0.00001). Both pooled HRs >1 indicated that FGFR gene amplification may be associated with poor OS in various cancers (Figures 3A and 3B). No evidence of heterogeneity was observed in the overall effects estimates with *I^2^* statistics of 0%. Four studies also reported disease-free survival (DFS) and *FGFR1* amplification, and the pooled result indicated that *FGFR1* amplification was also related to shorter DFS [HR 1.91 (95% CI: 1.43–2.54); *p*<0.0001; Figure S1].

### Sensitivity and publication bias

**Figure 4:**
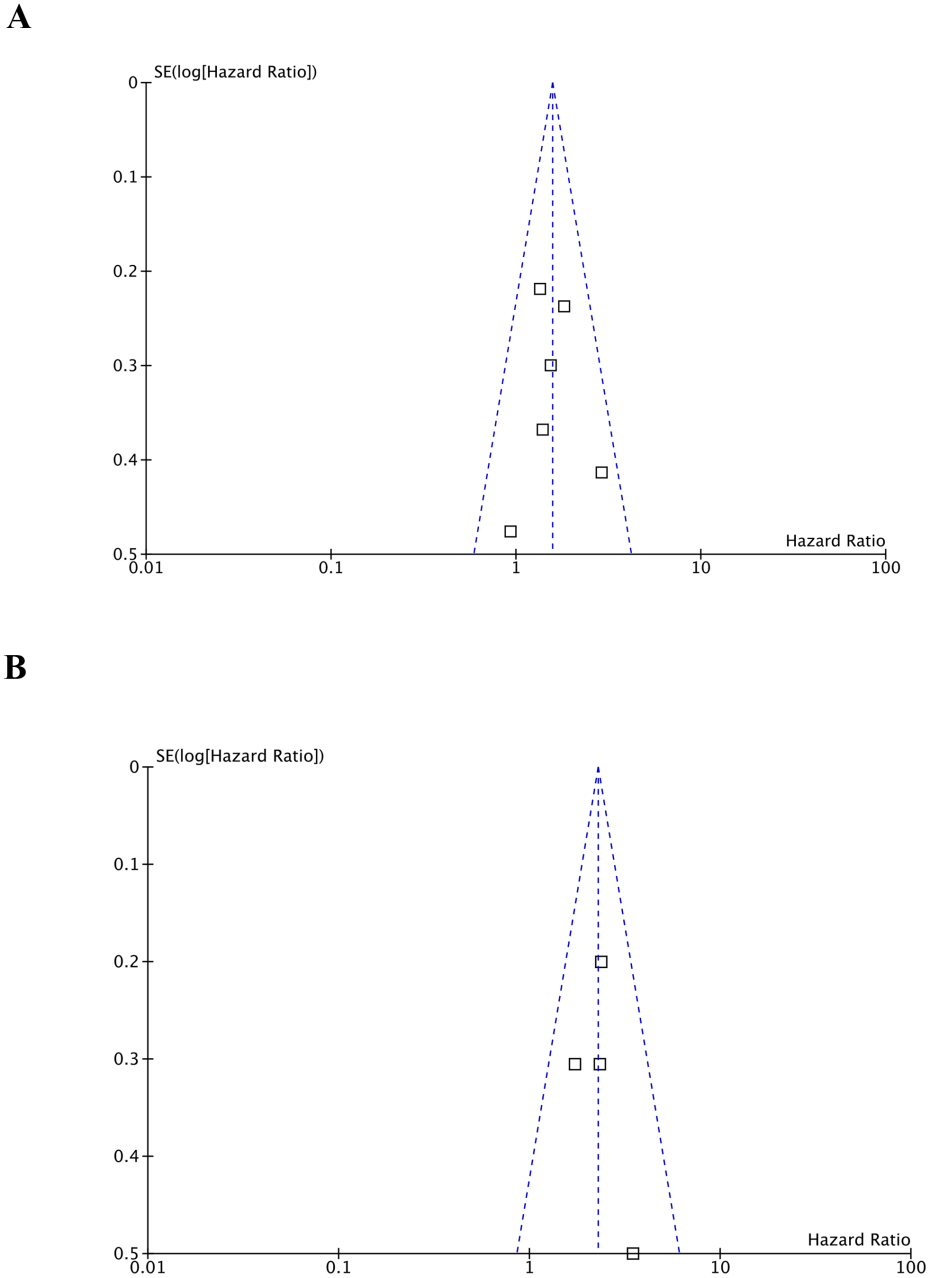
Funnel plots of the association between *FGFR* amplification and overall survival. (A) Publication bias for *FGFR1* amplification and overall survival in various cancers. (B) Publication bias for *FGFR2* amplification and overall survival in gastric cancer. Each point represents a separate study. Log [Hazard Ratio], natural logarithm of HR; SE, standard error.

The sensitivity analysis was performed by omitting one study at one time to measure its effect on the gene amplification prevalence and pooled HRs. Deletion of the study by Pros et al [14] significantly reduced the heterogeneity in the analysis of *FGFR2* amplification incidence. No other individual study influenced the results. Publication bias of the included studies was evaluated by Begg's funnel plots and Egger's tests, and it was only detected in the analysis of *FGFR1* amplification prevalence (*p* = 0.000 for Egger's test, Figure S2). In the other analyses, the Begg's funnel plots were almost symmetric and Egger's tests indicated that there was no evidence of publication bias (Figures 4A and 4B, Figure S3).

## Discussion

Deregulation of FGFR family signaling has been described in multiple cancers. Mechanisms of *FGFR* deregulation are included: 1) gene amplification (e.g. *FGFR1* amplification in lung cancer and breast cancer [4], [24], [29] and *FGFR2* amplification in gastric cancer [11], [31]); 2) gene mutation (e.g. *FGFR2* mutation in endometrial carcinomas [8] and *FGFR3* mutation in bladder cancer [3]); 3) gene translocation (e.g. *FGFR3* translocation in multiple myeloma [34]); 4) autocrine FGF signaling (e.g. FGF1 autocrine in ovarian cancer [35]). Compared to FGFR gene mutation and translocation, gene amplification of FGFR is most well-studied and associated with poor prognosis. To our knowledge, this is the first meta-analysis and systematic review on the association of FGFR gene amplification and cancer. In this article, we showed that *FGFR1* is amplified in lung cancer, breast cancer and, rarely, in pancreatic cancer and squamous cell cancer, whereas *FGFR2* amplification mainly occurs in gastric cancer and breast cancer. More importantly, we also performed this meta-analysis to assess the association between FGFR gene amplification and OS in different types of cancer.

As a new emerging therapy target, the FGFR gene has drawn much interest for developing specific inhibitors such as the multiple target inhibitors dovitinib, Ki23057, and ponatinib, and the highly selective inhibitors AZD4547 and BGJ398. Several preclinical studies have shown the striking therapeutic efficacy of AZD4547 and BGJ398 on FGFR gene–amplified cancers both *in vitro* and *in vivo* [15], [36], [37]. Some ongoing clinical trials have been summarized in a published paper [38]. Recently, a phase II study was designed to assess the activity of the FGFR inhibitor AZD4547 in patients with FGFR1- and FGFR2-amplified breast, squamous lung, or stomach cancers, whose cancers had progressed following previous chemotherapy (NCT01795768). Our data indicated that both FGFR1 and FGFR2 amplification were associated with poor survival in breast, lung, and gastric cancers. It is therefore reasonable to conduct more clinical trials that set FGFR copy number as an inclusion criterion. More importantly, our data highlighted the need for collaborative efforts in addressing FGFR as a therapeutic target. For example, the sample size for clinical trial evaluating anti-FGFR2 drug efficacy is about 400 (α = 5%, 1-β = 80%). According to our results, the incidence of FGFR2 amplification is 0.04, which is relatively low. As a result, over 10000 patients were needed to be enrolled in such clinical trail to identify a statistical difference.

Notably, various laboratory assays have been used to determine FGFR gene amplification. In situ hybridization techniques are used to measure gene amplification that relies on either fluorescence (FISH) or chromogenic and silver in situ hybridization (CISH and SISH). However, even using the same measurement method (e.g. FISH), different criteria have been used to define FGFR positivity. These differences in methodology can be the cause of the large range and heterogeneity of *FGFR1* amplification (from 2.6 to 30.9%). Standardization of the definition of ‘FGFR gene amplification’ is therefore urgently needed. As for other gene amplifications such as HER2 and EGFR, a scoring system is recommended for FGFR gene amplification. Nonetheless, most of the included studies in this meta-analysis used subjective scores without standardization. We believe that publication bias for *FGFR1* amplification prevalence is due to the many evaluation standards used. Despite this, the results from subgroup analysis relating to specific methodology (SNP screen, FISH, qPCR, and aCGH) were similar to those of the overall analysis (see Table S2).

In interpreting the results, some limitations of this meta-analysis should be addressed. First, we were unable to conduct stratified analysis based on possible confounders such as sex, *Helicobacter pylori* infection, smoking status, and alcohol intake due to insufficient data. Second, there was statistical heterogeneity among the studies regarding the prevalence of *FGFR1* amplification. Fortunately, we found that the heterogeneity may be due to the differences in validation standards. Third, publication bias among studies of *FGFR1* amplification may influence the results. Also, it is recommended that tests for funnel plot asymmetry should be used only when at least 10 studies are included in the meta-analysis [39].

## Conclusions

In conclusion, this meta-analysis and systematic review summarized the existing data on FGFR gene amplification and cancer outcomes. The results showed that patients with FGFR gene amplified cancers have shorter OS. Further studies with larger sample size and standardized scoring system are recommended to confirm this finding.

## Supporting Information

Figure S1 **Forest plots of studies evaluating HR of disease-free survivals comparing high** ***FGFR1*** **amplification and non-amplification.** The horizontal lines represent 95% CIs for estimating HR of FGFR1 amplification versus non-amplification in the meta-analysis. (▪) Overall estimates of the effects. CI, confidence interval; HR, harzard ratio.
(DOCX)

Figure S2 **Funnel plots of the prevalence of** ***FGFR*** **amplification.** A. Publication bias of the prevalence of *FGFR1* amplification. B. Publication bias of the prevalence of *FGFR2* amplification. Each point represents a separate study.
(DOCX)

Figure S3 **Funnel plots of the association between** ***FGFR*** **amplification and disease-free survival.** Each point represents a separate study. Log[Harzard Ratio],natural logarithm of HR. SE, standard error.
(DOCX)

Table S1 **FGFR gene amplification: characteristics of included studies.** *FGFR1*: fibroblast growth factor receptor 1; *FGFR2*: fibroblast growth factor receptor 2; NSCLC: non-small-cell lung cancer; SQLC: squamous cell lung cancer; OTSCC: oral tongue squamous cell carcinoma; FISH: fluorescence in situ hybridization; CISH: chromogenic in situ hybridization; SISH: silver in situ hybridization; qPCR: quantitative polymerase chain reaction; aCGH: assay comparative genomic hybridization; SNP: single-nucleotide polymorphism; N/A: not applicable.
(DOCX)

Table S2 **Overall and subgroup analysis of FGFR gene amplification prevalence.** *FGFR1*: fibroblast growth factor receptor 1; *FGFR2*: fibroblast growth factor receptor 2; NSCLC: non-small-cell lung cancer; SQLC: squamous cell lung cancer; OSCC: oral squamous cell carcinoma; OTSCC: oral tongue squamous cell carcinoma; FISH: fluorescence in situ hybridization; CISH: chromogenic in situ hybridization; SISH: silver in situ hybridization; PCR: polymerase chain reaction; aCGH: assay comparative genomic hybridization; SNP: single-nucleotide polymorphism; N/A: not applicable; PDAC: Pancreatic ductal adenocarcinoma.
(DOCX)

Checklist S1 **PRISMA statement.**
(DOC)
