# Supplementary material for: Prognostic Value of FGFR Gene Amplification in Patients with Different Types of Cancer: A Systematic Review and Meta-Analysis
Source: PLoS One. 2014 Aug 29;9(8):e105524. doi: 10.1371/journal.pone.0105524 (PMC4149366; doi:10.1371/journal.pone.0105524)
Supplement: Figure S1 — Forest plots of studies evaluating HR of disease-free survivals comparing high FGFR1 amplification and non-amplification. The horizontal lines represent 95% CIs for estimating HR of FGFR1 amplification versus non-amplification in the meta-analysis. (▪) Overall estimates of the effects. CI, confidence interval; HR, harzard ratio. (DOCX) [file pone.0105524.s001.docx]

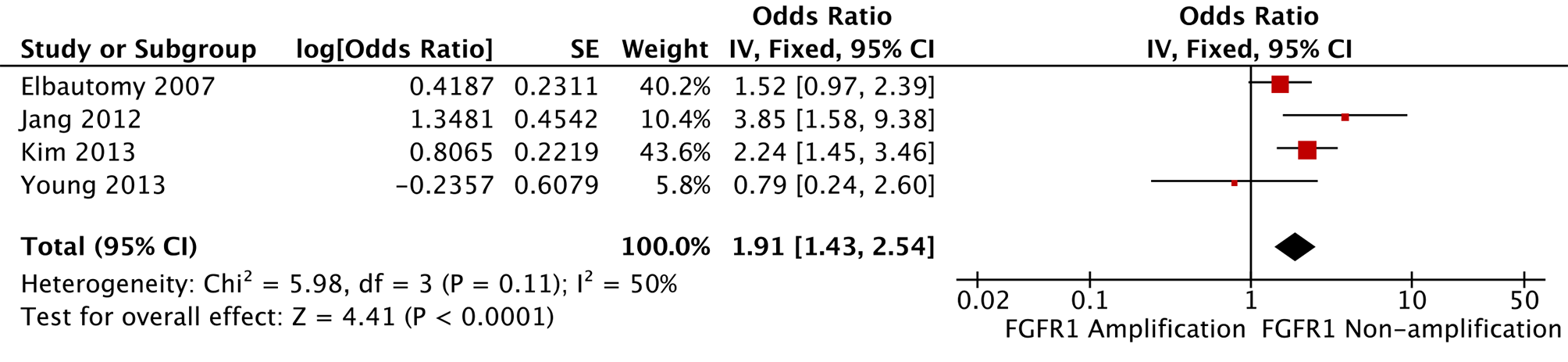


**Supplementary Figure 1. Forest plots of studies evaluating HR of disease-free survivals comparing high *FGFR1* amplification and non-amplification.**

The horizontal lines represent 95% CIs for estimating HR of FGFR1 amplification versus non-amplification in the meta-analysis.

(■) Overall estimates of the effects.

CI, confidence interval; HR, harzard ratio.
