# Supplementary material for: Prognostic Value of FGFR Gene Amplification in Patients with Different Types of Cancer: A Systematic Review and Meta-Analysis
Source: PLoS One. 2014 Aug 29;9(8):e105524. doi: 10.1371/journal.pone.0105524 (PMC4149366; doi:10.1371/journal.pone.0105524)
Supplement: Figure S2 — Funnel plots of the prevalence of FGFR amplification. A. Publication bias of the prevalence of FGFR1 amplification. B. Publication bias of the prevalence of FGFR2 amplification. Each point represents a separate study. (DOCX) [file pone.0105524.s002.docx]

**A**

**
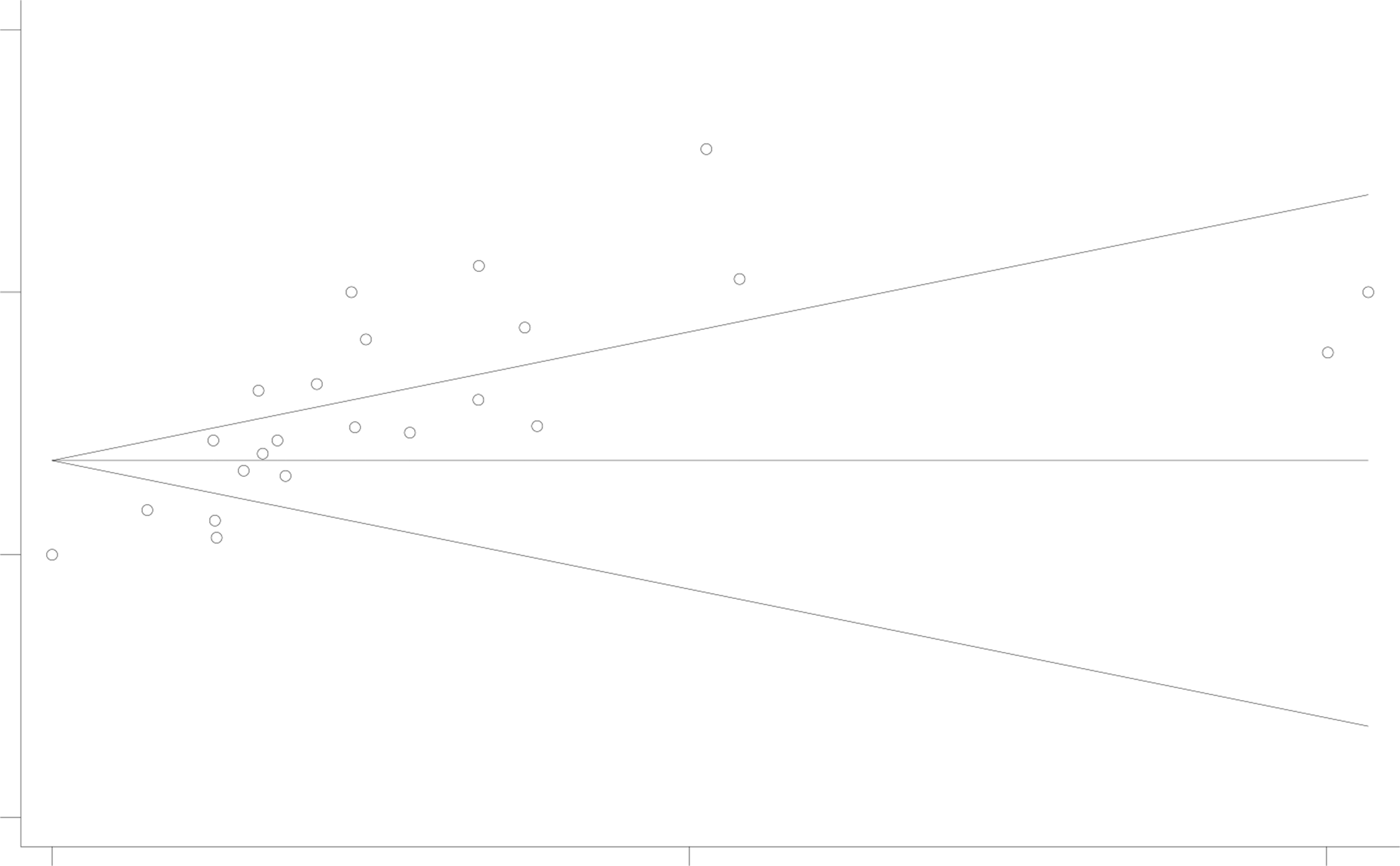
**

**B**

**
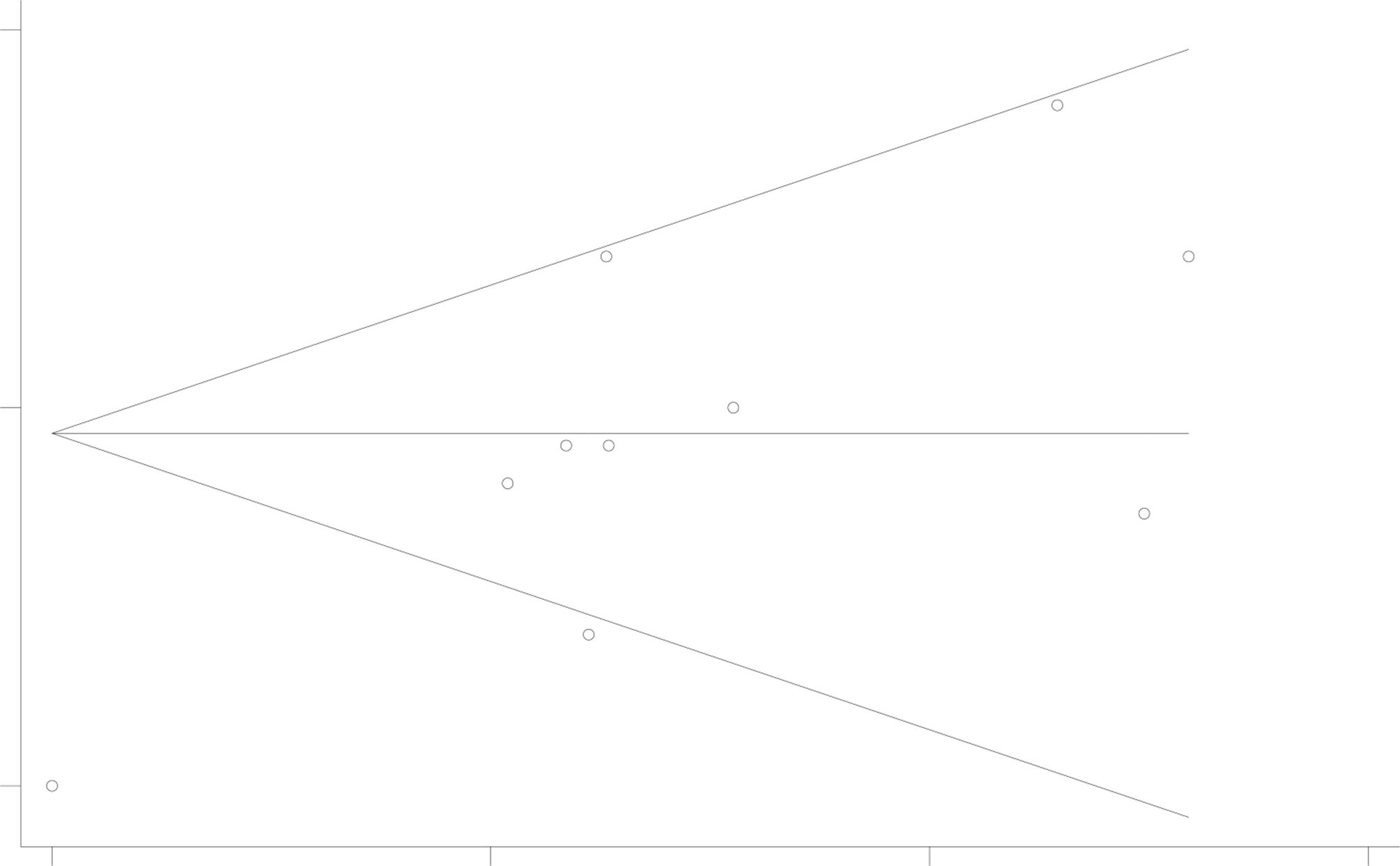
**

**Supplementary Figure 2. Funnel plots of the prevalence of *FGFR* amplification。**

A. Publication bias of the prevalence of *FGFR1* amplification. B. Publication bias of the prevalence of *FGFR2* amplification.

Each point represents a separate study.
