# Supplementary material for: Prognostic Value of FGFR Gene Amplification in Patients with Different Types of Cancer: A Systematic Review and Meta-Analysis
Source: PLoS One. 2014 Aug 29;9(8):e105524. doi: 10.1371/journal.pone.0105524 (PMC4149366; doi:10.1371/journal.pone.0105524)
Supplement: Figure S3 — Funnel plots of the association between FGFR amplification and disease-free survival. Each point represents a separate study. Log[Harzard Ratio],natural logarithm of HR. SE, standard error. (DOCX) [file pone.0105524.s003.docx]

**
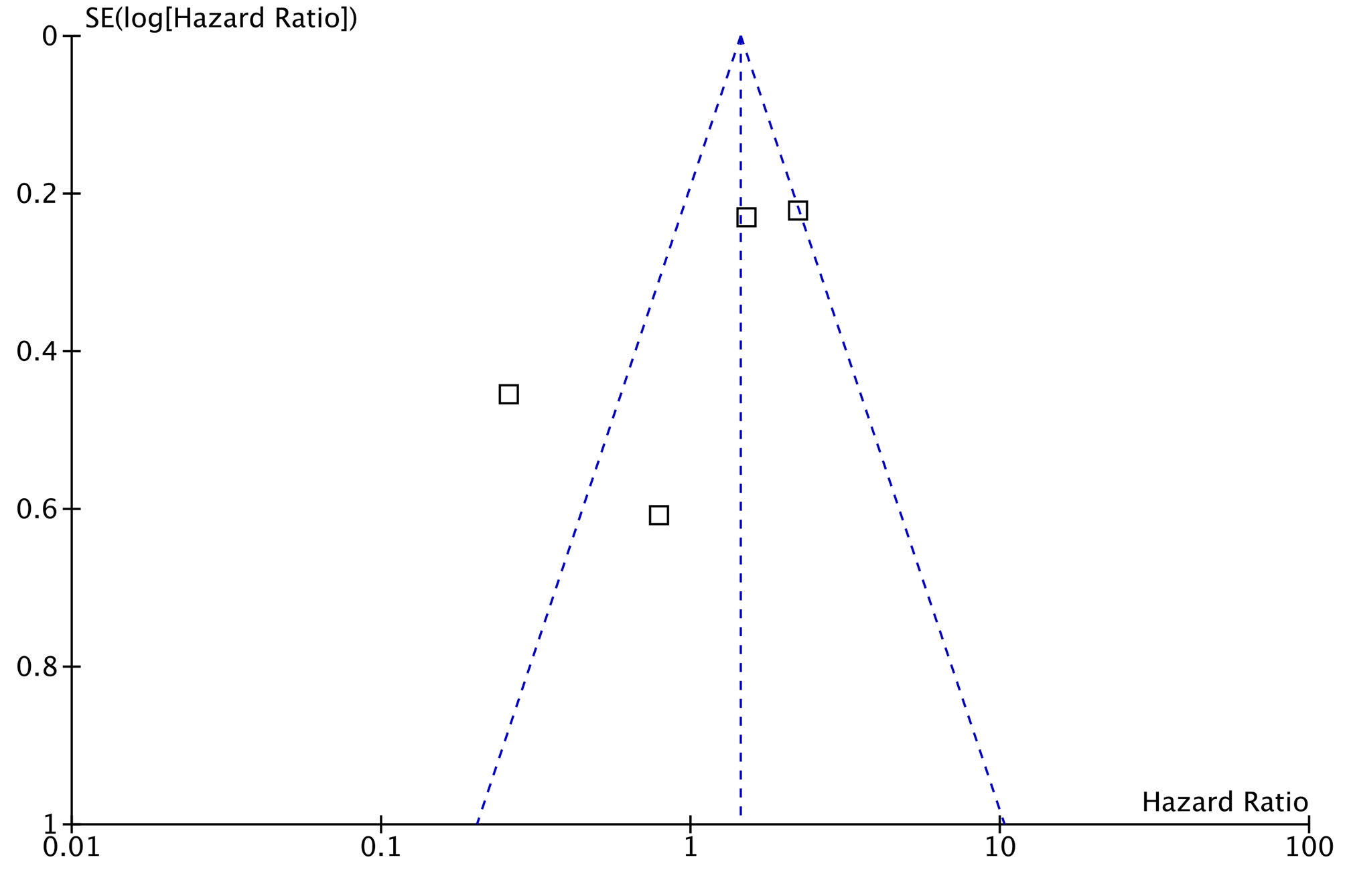
**

**Supplementary Figure 3. Funnel plots of the association between *FGFR* amplification and disease-free survival.**

Each point represents a separate study.

Log[Harzard Ratio],natural logarithm of HR

SE, standard error
