# Supplementary material for: Prognostic Value of FGFR Gene Amplification in Patients with Different Types of Cancer: A Systematic Review and Meta-Analysis
Source: PLoS One. 2014 Aug 29;9(8):e105524. doi: 10.1371/journal.pone.0105524 (PMC4149366; doi:10.1371/journal.pone.0105524)
Supplement: Table S1 — FGFR gene amplification: characteristics of included studies. FGFR1: fibroblast growth factor receptor 1; FGFR2: fibroblast growth factor receptor 2; NSCLC: non-small-cell lung cancer; SQLC: squamous cell lung cancer; OTSCC: oral tongue squamous cell carcinoma; FISH: fluorescence in situ hybridization; CISH: chromogenic in situ hybridization; SISH: silver in situ hybridization; qPCR: quantitative polymerase chain reaction; aCGH: assay comparative genomic hybridization; SNP: single-nucleotide polymorphism; N/A: not applicable. (DOCX) [file pone.0105524.s004.docx]

**Supplementary Table 1 FGFR gene amplification: characteristics of included studies**

| **Study** | **Cancer** | **Number of Patients** | **Country** | **Sex (Male/Female %)** | **Age (Years)** | | **Methods** | **Definition** | **Prevalence (%)** | **Prognosis** |
| --- | --- | --- | --- | --- | --- | --- | --- | --- | --- | --- |
| *FGFR1 amplification* | | | | | |  |  |  |  |  |
| Andre et al, 2013 [13] | Breast cancer | 81 | Switzerland | N/A | 56.5 | | SISH or PCR | ≥6 copy by qPCR | 30.9 | N/A |
| Brunello et al, 2012 [12] | Breast cancer | 15 | Italy | N/A | N/A | | CISH | Self-defined | 20 | N/A |
| Dutt et al, 2011 [20] | Lung squamous carcinoma | 57 | Multicenter | N/A | N/A | | SNP array | N/A | 21 | N/A |
|  | Lung adenocarcinoma | 588 | Multicenter | N/A | N/A | | SNP array | N/A | 3.4 | N/A |
| Elbauomy et al, 2007 [21] | Breast cancer | 496 | UK | N/A | N/A | | CISH | Self-defined | 8.7 | 478 survival data |
| Freier et al. 2007 [9] | Oral squamous cell carcinoma | 104 | Germany | N/A | N/A | | aCGH FISH | Self-defined | 17.3 | N/A |
| Heist et al, 2012 [3] | Lung squamous carcinoma | 226 | America | 68/55 | 67/69 | | FISH | *FGFR1/CEN8* ≥2.2 | 16.4 | Available |
| Jang et al, 2012 [22] | Ductal breast carcinoma in situ | 168 | Korea | N/A | 50 | | FISH | *FGFR1/CEN8* ≥2.2 | 6 | 422 survival data |
|  | Invasive breast carcinoma | 417 | Korea | N/A | 50 | | FISH | *FGFR1/CEN8* ≥2.2 | 12.5 | 422 survival data |
| Kim et al, 2013 [2] | Lung squamous carcinoma | 262 | Korea | 100/93 | 64/66 | | FISH | *FGFR1/CEN8* ≥9.0 | 13 | Available |
| Kohler et al, 2012 [23] | Lung cancer | 260 | Germany | N/A | N/A | | FISH | *FGFR1/CEN8* ≥4 | 7.7 | 117 survival data |
| Lee et al, 2011 [24] | Lung cancer | 61 | Korea | 82/100 | 61.4 | | FISH | Self-defined | 9.8 | Available |
| Lehnen et al, 2013 [14] | Pancreatic ductal adenocarcinoma | 155 | Germany | 2.58/58.06 | N/A | | FISH | *FGFR1/CEN8* ≥ 2 | 2.6 | 67 survival data |
| Pros et al, 2013 [17] | NSCLC | 265 | Spain | 85/100 | 64 | | FISH | Self-defined | 6.4 | N/A |
| Schildhaus et al, 2012 [25] | Lung squamous carcinoma | 290 | Germany | N/A | N/A | | FISH | Self-defined | 20 | N/A |
|  | Lung Adenocarcinoma | 97 | Germany | N/A | N/A | | FISH | Self-defined | 0 | N/A |
|  | Other lung cancer | 13 | Germany | N/A | N/A | | FISH | Self-defined | 15.4 | N/A |
| Turner et al, 2010 [13] | Breast cancer | 93 | UK | N/A | N/A | | CISH | Self-defined | 11.8 | N/A |
| Weiss et al, 2010 [4] | SQLC | 155 | Switzerland | N/A | N/A | | SNP array | N/A | 9.7 | Available |
| Weiss et al, 2010 [4] | Lung adenocarcinoma | 77 | Switzerland | N/A | N/A | | SNP array | N/A | 1.3 | N/A |
| Weiss et al, 2010 [4] | SQLC | 153 | Switzerland | N/A | N/A | | FISH | *FGFR1/CEN8* ≥ 2 | 22 | N/A |
| Young et al, 2013 [10] | OTSCC | 107 | Australia | 90/65 | 57.5/56 | | FISH | *FGFR1/CEN8* ≥ 2 | 9.3 | A |
| Zhang et al, 2012 [28] | Lung cancer | 254 | China | 12/88 | N/A | | FISH | *FGFR1/CEN8* ≥ 2 | 8.7 | N/A |
| *FGFR2 amplification* | | | | | | | | | | |
| Deng et al, 2012 [6] | Gastric cancer | 156 | Singapore | N/A | N/A | | SNP array for screen  PCR and FISH for validation (high *FGFR2* qPCR values only) |  | 9-10 | N/A |
| Jung et al, 2012 [15] | Gastric cancer | 313 | Korea | 50/74.2 | N/A | | FISH | *FGFR2/CEP-10* ≥2.0 | 4.5 | A |
| Kilgour 2012 [28] | Gastric cancer | 408 | Caucasian population | N/A | N/A | | FISH | *FGFR2/CEP-10* ≥2.0 | 7 | A |
| Kilgour 2012,  [28] | Gastric cancer | 356 | Korea | N/A | N/A | | FISH | *FGFR2/CEP-10* ≥2.0 | 4 | A |
| Matsumoto et al, 2012 [5] | Gastric cancer | 267 | Japan | 100/68 | 67/63 | | PCR FISH | *FGFR2/CEP-10* ≥2.0 | 4.1 | A |
| Pros et al, 2013 [17] | NSCLC | 266 | Spain | 85/100 | 64 | | FISH | Self-defined | 0 | N/A |
| Turner 2010  [13] | Breast cancer | 56 | The Netherlands | N/A | N/A | | aCGH | CGH logRatio  >0.45 | 3.6 | N/A |
| Xie et al, 2013 [27] | Gastric cancer | 131 | Beijing, China | 71 | 62 | | aCGH | CGH logRatio  >0.8 | 2 | N/A |
| Xie et al, 2013 [27] | Gastric cancer | 197 | Shanghai, China | 67.5 | 62 | | FISH | *FGFR2/CEP-10* ≥2.0 | 5 | N/A |
| Xie et al, 2013 [27] | Gastric cancer | 97 | Caucasian population | 68 | 67 | | FISH | *FGFR2/CEP-10* ≥2.0 | 7 | N/A |

*FGFR1*: fibroblast growth factor receptor 1; *FGFR2*: fibroblast growth factor receptor 2; NSCLC: non-small-cell lung cancer; SQLC: squamous cell lung cancer; OTSCC: oral tongue squamous cell carcinoma; FISH: [fluorescence in situ hybridization](http://www.google.com.hk/search?newwindow=1&safe=strict&biw=1366&bih=582&q=fluorescence+in+situ+hybridization&revid=1475705252&sa=X&ei=3II0UtiJA8PKkQWJmICYCw&ved=0CH4Q1QIoAA); CISH: chromogenic in situ hybridization; SISH: silver in situ hybridization; qPCR: quantitative polymerase chain reaction; aCGH: assay comparative genomic hybridization; SNP: single-nucleotide polymorphism; N/A: not applicable.
