# Supplementary material for: Prognostic Value of FGFR Gene Amplification in Patients with Different Types of Cancer: A Systematic Review and Meta-Analysis
Source: PLoS One. 2014 Aug 29;9(8):e105524. doi: 10.1371/journal.pone.0105524 (PMC4149366; doi:10.1371/journal.pone.0105524)
Supplement: Table S2 — Overall and subgroup analysis of FGFR gene amplification prevalence. FGFR1: fibroblast growth factor receptor 1; FGFR2: fibroblast growth factor receptor 2; NSCLC: non-small-cell lung cancer; SQLC: squamous cell lung cancer; OSCC: oral squamous cell carcinoma; OTSCC: oral tongue squamous cell carcinoma; FISH: fluorescence in situ hybridization; CISH: chromogenic in situ hybridization; SISH: silver in situ hybridization; PCR: polymerase chain reaction; aCGH: assay comparative genomic hybridization; SNP: single-nucleotide polymorphism; N/A: not applicable; PDAC: Pancreatic ductal adenocarcinoma. (DOCX) [file pone.0105524.s005.docx]

**Supplementary Table 2 Overall and subgroup analysis of FGFR gene amplification prevalence**

| **Subgroup** | **No. of Datasets** | **Incidence** | **95% Confidence Interval** | ***p* Value for Heterogeneity** | **I^2^ (%)** | **Model** | ***p* Value for Publication Bias** |
| --- | --- | --- | --- | --- | --- | --- | --- |
| *FGFR1 amplification* | | | | | | | |
| Overall | 23 | 0.11 | 0.08–0.13 | 0.000 | 91.3 | Random | 0.000 |
| Cancer type |  |  |  |  |  |  |  |
| Lung | 14 | 0.09 | 0.06–0.13 | 0.000 | 92.0 | Random |  |
| Breast | 6 | 0.12 | 0.08–0.17 | 0.000 | 80.8 | Random |  |
| PDAC | 1 | 0.17 | 0.10–0.25 |  |  |  |  |
| OTSCC | 1 | 0.09 | 0.04–0.15 |  |  |  |  |
| OSCC | 1 | 0.03 | 0.00–0.05 |  |  |  |  |
| Ethnicity |  |  |  |  |  |  |  |
| Caucasian | 16 | 0.11 | 0.08–0.15 | 0.000 | 92.5 | Random |  |
| Asian | 5 | 0.10 | 0.07–0.13 | 0.044 | 59.1 | Random |  |
| Multicenter | 2 | 0.11 | -0.06–0.29 | 0.001 | 90.4 | Random |  |
| Methods |  |  |  |  |  |  |  |
| FISH | 15 | 0.10 | 0.07–0.14 | 0.000 | 92.6 | Random |  |
| CISH | 3 | 0.09 | 0.07–0.12 | 0.396 | 0.0 | Fixed |  |
| SNP Array | 4 | 0.06 | 0.02–0.11 | 0.000 | 85.2 | Random |  |
| SISH or PCR | 1 | 0.31 | 0.21–0.41 |  |  |  |  |
| *FGFR2 amplification* | | | | | | | |
| Overall | 10 | 0.04 | 0.02–0.06 | 0.000 | 83.5 | Random | 0.248 |
| Ethnicity |  |  |  |  |  |  |  |
| Caucasian | 4 | 0.06 | 0.00–0.09 | 0.000 | 89.8 | Random |  |
| Asian | 6 | 0.04 | 0.03–0.06 | 0.154 | 37.9 | Fixed |  |
| Methods |  |  |  |  |  |  |  |
| FISH | 6 | 0.04 | 0.02–0.07 | 0.000 | 88.5 | Random |  |
| aCGH | 2 | 0.02 | 0.00–0.04 | 0.104 | 39.5 | Fixed |  |
| SNP array | 1 | 0.09 | 0.05–0.13 |  |  |  |  |
| PCR or FISH | 1 | 0.04 | 0.02–0.07 |  |  |  |  |

*FGFR1*: fibroblast growth factor receptor 1; *FGFR2*: fibroblast growth factor receptor 2; NSCLC: non-small-cell lung cancer; SQLC: squamous cell lung cancer; OSCC: oral squamous cell carcinoma; OTSCC: oral tongue squamous cell carcinoma; FISH: [fluorescence in situ hybridization](http://www.google.com.hk/search?newwindow=1&safe=strict&biw=1366&bih=582&q=fluorescence+in+situ+hybridization&revid=1475705252&sa=X&ei=3II0UtiJA8PKkQWJmICYCw&ved=0CH4Q1QIoAA); CISH: chromogenic in situ hybridization; SISH: silver in situ hybridization; PCR: polymerase chain reaction; aCGH: assay comparative genomic hybridization; SNP: single-nucleotide polymorphism; N/A: not applicable; PDAC: Pancreatic ductal adenocarcinoma
